# Diagnostic delay is common for patients with axial spondyloarthritis: results from the National Early Inflammatory Arthritis Audit

**DOI:** 10.1093/rheumatology/keab428

**Published:** 2021-05-12

**Authors:** Mark D Russell, Fiona Coath, Mark Yates, Katie Bechman, Sam Norton, James B Galloway, Joanna Ledingham, Raj Sengupta, Karl Gaffney

**Affiliations:** Centre for Rheumatic Diseases, King’s College London, London; Rheumatology Department, Norfolk and Norwich University Hospital, Norwich; Centre for Rheumatic Diseases, King’s College London, London; Centre for Rheumatic Diseases, King’s College London, London; Centre for Rheumatic Diseases, King’s College London, London; Centre for Rheumatic Diseases, King’s College London, London; Rheumatology Department, Portsmouth Hospitals University NHS Trust, Portsmouth; Department of Rheumatology, Royal National Hospital for Rheumatic Diseases, Bath, UK; Rheumatology Department, Norfolk and Norwich University Hospital, Norwich

**Keywords:** axial spondyloarthritis, ankylosing spondylitis, diagnosis, delay, NEIAA, national audit, EIA, rheumatoid arthritis

## Abstract

**Objectives:**

Updated guidelines for patients with axial SpA (axSpA) have sought to reduce diagnostic delay by raising awareness among clinicians. We used the National Early Inflammatory Arthritis Audit (NEIAA) to describe baseline characteristics and time to diagnosis for newly referred patients with axSpA in England and Wales.

**Methods:**

Analyses were performed on sociodemographic and clinical metrics, including time to referral and assessment, for axSpA patients (*n* = 784) recruited to the NEIAA between May 2018 and March 2020. Comparators were patients recruited to the NEIAA with RA (*n* = 9270) or mechanical back pain (MBP; *n* = 370) in the same period.

**Results:**

Symptom duration prior to initial rheumatology assessment was longer in axSpA than RA patients (*P* < 0.001) and non-significantly longer in axSpA than MBP patients (*P* = 0.062): 79.7% of axSpA patients had symptom durations of >6 months, compared with 33.7% of RA patients and 76.0% of MBP patients. Following referral, the median time to initial rheumatology assessment was longer for axSpA than RA patients (36 *vs* 24 days; *P* < 0.001) and similar to MBP patients (39 days; *P* = 0.30). Of the subset of patients deemed eligible for early inflammatory arthritis pathway follow-up, fewer axSpA than RA patients had disease education provided (77.5% *vs* 97.8%) and RA patients reported a better understanding of their condition and treatment.

**Conclusion:**

Diagnostic delay in axSpA remains a major challenge despite improved disease understanding and updated referral guidelines. Disease education is provided to fewer axSpA than RA patients, highlighting the need for specialist clinics and support programmes for axSpA patients.

**Rheumatology key messages**

- Diagnostic delay remains a challenge in axSpA and should be a focus for service development.
- MSK-HQ scores suggest that the functional impact of axSpA is no less than for RA.
- Patient education and empowerment remain an unmet need in axSpA.

## Introduction

Axial SpA (axSpA) is a chronic inflammatory disease characterized by inflammation of the sacroiliac joints and spine, peripheral arthritis, enthesitis, dactylitis and extraskeletal manifestations, including acute anterior uveitis, inflammatory bowel disease and psoriasis. Diagnostic delay is a significant problem in axSpA [1, 2]. A meta-analysis of 64 studies in axSpA patients reported a pooled mean diagnostic delay of 6.7 years [3]. Delayed diagnosis in axSpA is associated with worse clinical, humanistic and economic outcomes [4], while treatment with TNF inhibitors improves clinical outcomes and radiographic progression more effectively when commenced earlier in the disease process [5].

International guidelines have been published to inform referral pathways, with the aim of reducing diagnostic delay in axSpA [6–8]. Whether increased clinician awareness through publication of guidelines and enhanced access to diagnostic imaging has translated into reduced diagnostic delay for patients newly referred with axSpA is not known.

In this study we used the National Early Inflammatory Arthritis Audit (NEIAA) to describe baseline sociodemographic and clinical characteristics, including time to diagnosis, for patients with axSpA in England and Wales between May 2018 and March 2020.

## Methods

### Study sample

The NEIAA captures data on patients referred to rheumatology services in England and Wales with suspected early inflammatory arthritis (EIA) [9, 10]. Its primary purpose is to measure care quality across healthcare providers, to enable benchmarking and to stimulate quality improvement activity. Since 8 May 2018, providers of rheumatology services in the National Health Service (NHS; see the glossary in the supplementary material, available at *Rheumatology* online) in England and Wales have been requested to submit data to the NEIAA on patients ≥16 years of age newly referred with suspected inflammatory arthritis, regardless of the ultimate diagnosis; this includes referrals of patients from primary care clinicians, musculoskeletal triage services and secondary care specialties. The catchment population for the NEIAA is explicitly defined to include any inflammatory arthritis, including patients with suspected or confirmed axSpA, with or without peripheral joint involvement. Further interpretation of this definition is at the discretion of the clinical units.

In this study we included all patients enrolled in the NEIAA seen between 8 May 2018 and 1 March 2020 and diagnosed with axSpA by their treating rheumatologist. Comparators were all patients in the NEIAA who received a diagnosis of RA or mechanical back pain (MBP) during the same study period. Psoriatic arthritis is encoded as a separate diagnosis in the NEIAA and was not included in these analyses.

Clinician-reported metrics collected at baseline in the NEIAA for all patients were included as follows: age, gender, ethnicity (White, Black British/African/Caribbean, Asian/Asian British, mixed/other ethnic groups), smoking status (current smoker, ex-smoker, never smoker), work status (patients 16–65 years of age in paid work >20 h/week), symptom duration (defined as the duration of symptoms prior to referral; recorded in the NEIAA as an ordered categorical variable: <1 month, 1–3 months, 3–6 months, 6–12 months, 1–5 years, 5–10 years, >10 years), time to initial rheumatology assessment (calculated from the date of receipt of referral to the first rheumatology assessment) and index of multiple deprivation (IMD; an area-level composite score of socio-economic position; see the glossary in the supplementary material, available at *Rheumatology* online for further information) [11]. For axSpA patients, baseline data on HLA-B27 status and the presence of sacroiliitis/SpA on radiographs and/or MRI were presented, where available. Comprehensive details of the data collection methodology are available in the NEIAA annual report [9, 10].

Although the focus of axSpA data in the NEIAA is the initial presentation, a subset of patients recruited to the NEIAA are deemed eligible for more frequent follow-up within an EIA pathway by the treating rheumatologist; the NEIAA relies upon the clinician’s opinion as to whether it is appropriate to enrol a patient into an EIA pathway. Clinicians are specifically advised to include (but not limit to) patients who are going to receive disease-modifying treatment [9, 10]. For EIA-eligible patients, additional clinical data are collected and recorded in the NEIAA by the clinician, including baseline tender joint count (TJC; 0–28 joints), swollen joint count (SJC; 0–28 joints), patient-reported global health score (0–100 scale, from best to worst), ESR (mm/h) and/or CRP (mg/l), initial DMARD treatment commenced (if any; patients could be commenced on more than one DMARD simultaneously) and whether disease-specific educational information (printed or online material; clinician reported) has been provided to patients. For EIA-eligible patients, questionnaires are used to collect the following patient-reported outcome measures (PROMs): HAQ Disability Index (HAQ-DI; see the glossary in the supplementary material, available at *Rheumatology* online for further information), Musculoskeletal Health Questionnaire (MSK-HQ; see the glossary in the supplementary material, available at *Rheumatology* online for further information) [12] and Work Productivity and Activity Index (WPAI) overall impairment, which incorporates absenteeism (numbers of hours of work missed as a percentage of the total hours worked) and presenteeism (degree to which patients’ health affects their productivity at work) (see the glossary in the supplementary material, available at *Rheumatology* online for further information) [10, 13].

### Statistical analyses

Data were presented as medians and interquartile ranges for continuous measures and absolute counts and percentages for categorical measures. Due to the large sample sizes, *P*-values were not presented for comparisons of demographic characteristics to avoid statistical inferences based on small differences between groups.

Statistical comparisons were performed to test the following primary hypotheses (the statistical test used is shown in parentheses): symptom duration prior to referral would be longer for axSpA than RA patients (Pearson’s chi-squared test), median time to assessment in a rheumatology clinic following referral would be longer for axSpA than RA patients (Kruskal–Wallis test) and the proportion of patients with axSpA or RA assessed within 3 weeks of referral would have improved since the launch of the NEIAA in 2018 (linear mixed model, with the assumption of a linear relationship). Differences were considered statistically significant for *P*-values <0.05.

Additional exploratory analyses were performed to describe the following: symptom duration for male *vs* female axSpA patients, assessed using logistic regression; median HAQ-DI, MSK-HQ and WPAI overall impairment in EIA-eligible axSpA and RA patients; provision of disease-specific education in EIA-eligible axSpA *vs* EIA-eligible RA patients (mean difference and 95% CIs calculated using Student’s *t*-test) and patients’ understanding of their condition/treatment and confidence in managing their symptoms; DASs (median TJC, SJC, patient global assessment score, ESR and CRP) in EIA-eligible axSpA and RA patients; and the relationship between TJC, SJC and whether DMARDs were commenced in EIA-eligible axSpA patients, assessed using logistic regression. Results were described in the text without *P*-values, recognizing the exploratory nature of these comparisons. Where logistic regression was used, results were presented as odds ratios (ORs) with 95% CIs.

Statistical analyses were performed using Stata version 16.1 (StataCorp, College Station, TX, USA).

Approval to conduct this research using the NEIAA dataset was obtained from the Healthcare Quality Improvement Partnership. No informed patient consent was required, as this dataset was created from routinely collected data during clinical practice. Data access requests can be made through the Healthcare Quality Improvement Partnership.

## Results

### Baseline characteristics of patients with axSpA compared with RA and MBP

A total of 784 patients with axSpA, 9270 patients with RA and 370 patients with MBP had data available (Table 1). The axSpA patients were younger (38 years) than the RA patients (61 years) and of similar age to the MBP patients (40 years). More axSpA patients were male (59.9%) than RA (36.6%) or MBP (40.8%) patients. Ethnicities were similar between cohorts, with White ethnicity being the most common (86.6% axSpA, 86.8% RA, 81.6% MBP). The axSpA patients were more likely to be current smokers (26.5%) than RA (21.8%) or MBP (21.7%) patients. A total of 19.4% of axSpA patients were within the most-deprived IMD quintile compared with 20.7% of RA and 21.6% of MBP patients. Baseline data on HLA-B27 status, radiographic status and MRI status were available for 55.7%, 51.8% and 52.0% of axSpA patients, respectively. Of the axSpA patients with data available, 59.5% were HLA-B27 positive, 45.8% had radiographic sacroiliitis/SpA and 86.3% had sacroiliitis/SpA on MRI. Of the 752 axSpA patients between the ages of 16 and 65 years with data available on work participation, 586 (77.9%) were in paid work for >20 h/week compared with 3666/5558 (66.0%) RA patients and 231/327 (70.6%) MBP patients.

**Table 1:** Baseline characteristics of patients with axSpA, RA and MBP

Characteristics	axSpA (*n* = 784)	RA (*n* = 9270)	MBP (*n* = 370)
Age, years, median (IQR)	38 (30–49)	61 (49–71)	40 (30–49)
Gender, *n* (%)			
Female	314 (40)	5877 (63)	219 (59)
Male	470 (60)	3393 (37)	151 (41)
Ethnicity, *n* (%)			
White	676 (87)	7971 (87)	297 (82)
Black British/African/Caribbean	11 (1)	267 (3)	11 (3)
Asian/Asian British	54 (7)	639 (7)	35 (10)
Mixed/Other Ethnic Groups	40 (5)	306 (3)	21 (6)
Not known	3	87	6
Smoking status, *n* (%)			
Current smoker	188 (27%)	1879 (22%)	68 (22%)
Ex-smoker	152 (21%)	2772 (32%)	61 (20%)
Never smoked	370 (52%)	3984 (46%)	184 (59%)
Not known	74	635	57
Within most-deprived IMD quintile, *n* (%)			
No	570 (81)	6808 (79)	250 (78)
Yes	137 (19)	1781 (21)	69 (22)
Not known	77	681	51
Duration of symptoms, *n* (%)			
<1 month	16 (2)	767 (8)	10 (3)
1–3 months	67 (9)	3149 (34)	35 (10)
3–6 months	75 (10)	2177 (24)	38 (11)
6–12 months	114 (15)	1640 (18)	68 (20)
1–5 years	253 (33)	1135 (12)	110 (32)
5–10 years	121 (16)	175 (2)	47 (14)
>10 years	133 (17)	142 (2)	38 (11)
Not known	5	85	24
Time to initial assessment, days, median (IQR)	36 (20–64)	24 (14–44)	39 (21–71)
Assessment within 3 weeks of referral, *n* (%)			
No	548 (70)	5053 (55)	267 (73)
Yes	231 (30)	4128 (45)	101 (27)
Not known	5	8	2

Missing data are shown but not are included within the percentages for each category. IQR: interquartile range.

### Symptom duration and assessment times for patients with axSpA compared with RA and MBP

**Fig. 1:**
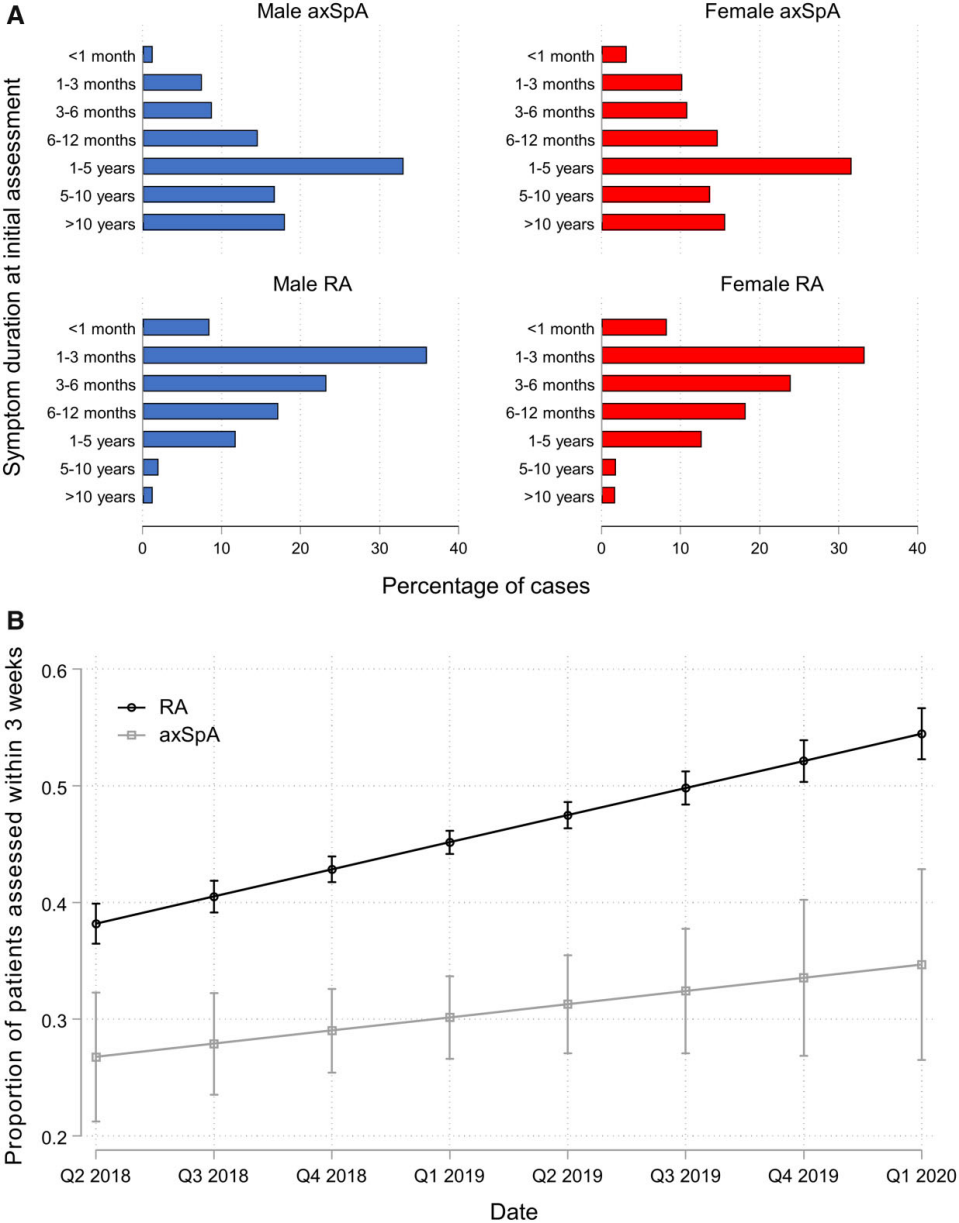
Diagnostic delay for axSpA and RA patients in the NEIAA (**A**) Symptom duration prior to referral for initial rheumatology assessment for axSpA *vs* RA patients, separated by gender. (**B**) Change over time in the proportion of axSpA and RA patients assessed in a rheumatology clinic within 3 weeks of referral using a linear mixed model.

The duration of symptoms prior to referral was substantially longer for axSpA than RA patients (*P* < 0.001), as shown in Fig. 1A and Table 1. There was a trend towards longer symptom duration in axSpA than MBP patients, although not statistically significant (*P* = 0.062). Of the 779 axSpA patients with data available on symptom duration, 621 (79.7%) had symptom durations of >6 months, compared with 3092/9185 (33.7%) RA patients and 263/346 (76.0%) MBP patients. A total of 32.6% of axSpA patients had experienced symptoms for >5 years compared with 3.5% of RA patients and 24.6% of MBP patients. In patients with axSpA, male gender associated with symptom durations of >6 months [OR 1.50 (95% CI 1.06, 2.10)]; for symptom durations of >5 years, differences between male and female axSpA patients were less apparent [OR 1.28 (95% CI 0.94, 1.74)].

The median time from referral to initial rheumatology assessment was longer for axSpA (36 days) than RA patients (24 days; *P* < 0.001) and similar to MBP patients (39 days; *P* = 0.30). The proportion of axSpA patients assessed in a rheumatology clinic within 3 weeks of referral increased from 26.7% in May 2018 to 34.7% in March 2020, while the proportion of RA patients assessed within 3 weeks of referral increased from 38.2% in May 2018 to 54.5% in March 2020 (Fig. 1B). Most axSpA patients (72.4%) were referred by primary care clinicians, 14.1% were referred by musculoskeletal triage services, 1.9% by gastroenterology, 1.4% by ophthalmology, 0.4% by dermatology and 9.8% from other sources.

### Comparison of EIA-eligible and EIA-ineligible axSpA patients

A subset of axSpA patients in the NEIAA were deemed eligible for more frequent follow-up in an EIA pathway by the treating rheumatologists. Of 762 axSpA patients with data available on EIA eligibility, 222 (29.1%) were eligible for EIA follow-up compared with 8780/9244 (95.0%) RA patients. Baseline characteristics were similar between EIA-eligible and EIA-ineligible axSpA patients (Supplementary Table S1, available at *Rheumatology* online). For EIA-eligible axSpA (axSpA-EIA) and EIA-eligible RA (RA-EIA) patients, additional clinical data were collected in the NEIAA, including PROMs (Table 2), DASs and DMARDs initiated (Table 3).

**Table 2:** PROMs at baseline for EIA-eligible patients with axSpA and RA

PROMs	EIA-eligible axSpA (*n* = 222)	EIA-eligible RA (*n* = 8780)
HAQ-DI, median (IQR)	0.8 (0.5,1.4)	1.1 (0.6,1.7)
MSK-HQ, median (IQR)	25 (17,34)	24 (16,33)
WPAI overall impairment, %, median (IQR)	32 (20,53)	30 (10,50)
Absenteeism, %, median (IQR)	0 (0,0)	0 (0,25)
Presenteeism, %, median (IQR)	40 (20,60)	50 (20,70)
Patient education within a month of diagnosis, *n* (%)		
No	45 (22%)	192 (2%)
Yes	155 (78%)	8,400 (98%)
Not known	22	188
Understanding of your condition/treatment, *n* (%)		
Not at all	19 (18%)	267 (8%)
Slightly	31 (30%)	645 (19%)
Moderately	30 (29%)	1193 (35%)
Very well	18 (17%)	1091 (32%)
Completely	6 (6%)	225 (7%)
Not known	118	5359
Confidence in managing your symptoms, *n* (%)		
Not at all	12 (11%)	402 (12%)
Slightly	35 (33%)	826 (24%)
Moderately	33 (31%)	1285 (38%)
Very	18 (17%)	746 (22%)
Extremely	7 (7%)	156 (5%)
Not known	117	5365

Missing data are shown but are not included within the percentages for each category. IQR: interquartile range.

**Table 3:** Disease activity scores at baseline and DMARD use by 3 months for EIA-eligible patients with axSpA and RA

Characteristics	EIA-eligible axSpA (*n* = 222)	EIA-eligible RA (*n* = 8780)
Baseline TJC, median (IQR)	0 (0–2)	6 (3–12)
Baseline SJC, median (IQR)	0 (0–1)	5 (2–9)
Baseline patient global assessment, median (IQR)	50 (10–70)	60 (40–80)
Baseline CRP, mg/l, median (IQR)	5 (2–17)	12 (4–30)
Baseline ESR, mm/h, median (IQR)	8 (2–26)	27 (12–44)
DMARD therapy commenced by 3 months, *n* (%)		
No	45 (54)	231 (3)
Yes	38 (46)	7499 (97)
Not known	139	1050
MTX commenced by 3 months, *n* (%)		
No	144 (92)	2242 (30)
Yes	12 (8)	5343 (70)
Not known	66	1195
SSZ commenced by 3 months, *n* (%)		
No	147 (95)	5923 (87)
Yes	8 (5)	900 (13)
Not known	67	1957
HCQ commenced by 3 months, *n* (%)		
No	149 (97)	4623 (65)
Yes	5 (3)	2493 (35)
Not known	68	1664
Other DMARD commenced by 3 months, *n* (%)		
No	143 (91)	6632 (100)
Yes	14 (9)	25 (0)
Not known	65	2123

Missing data are shown but are not included within the percentages for each category. IQR: interquartile range.

### PROMs at baseline in EIA-eligible axSpA and RA patients

The median HAQ-DI scores were lower at baseline in axSpA-EIA than RA-EIA patients (0.8 *vs* 1.1, respectively), whereas the median MSK-HQ scores were similar (25 *vs* 24, respectively). In both cohorts, the burden of disease was substantial across the 14 domains comprising MSK-HQ (Supplementary Fig. S1, available at *Rheumatology* online). For patients 16–65 years of age, the median WPAI overall impairment was greater, albeit modestly, for axSpA-EIA than RA-EIA patients (32.2% *vs* 30.0%, respectively).

Fewer axSpA-EIA than RA-EIA patients had disease-specific education provided within 1 month of diagnosis [77.5% *vs* 97.8%, respectively; mean difference 20.3% (95% CI 18.0, 22.5)]. The axSpA-EIA patients were less likely than RA-EIA patients to report having understood their condition and treatment very well or completely (23% *vs* 39%, respectively), while 24% of axSpA-EIA patients and 27% of RA-EIA patients felt very or extremely confident in managing their symptoms.

### DASs at baseline and DMARD use by 3 months in EIA-eligible axSpA and RA patients

RA-EIA patients had higher median TJCs (6 *vs* 0), SJCs (5 *vs* 0), patient global assessment scores (60 *vs* 50), CRP (12 *vs* 5 mg/l) and ESR (27 *vs* 8 mm/h) than axSpA-EIA patients. Data on the BASDAI score or Ankylosing Spondylitis Disease Activity Score (ASDAS) were not available, as collection of these measures were not core aims of the NEIAA.

Of 7730 RA-EIA patients with data available, 7499 (97.0%) commenced a DMARD within 3 months of referral; 5343 commenced MTX, 900 commenced SSZ and 2493 commenced HCQ. Of 83 axSpA-EIA patients, 38 (45.8%) patients with data available commenced a DMARD by 3 months; 12 commenced MTX, 8 commenced SSZ, 5 commenced HCQ and 14 commenced other unspecified DMARDs for which further details were unavailable. The axSpA-EIA patients were more likely to commence DMARDs if they had higher TJCs [OR 1.53 (95% CI 1.17, 2.01)] or higher SJCs [OR 2.07 (95% CI 1.28–3.36)].

## Discussion

In this study we described the characteristics of patients newly referred with axSpA in England and Wales between May 2018 and March 2020 using the NEIAA dataset. We showed that diagnostic delay remains a major challenge in axSpA, despite improved understanding of this disease and updated referral guidelines [6–8, 14]. Eighty percent of axSpA patients reported symptom durations of >6 months at initial assessment and one-third reported symptoms of >5 years. The time to initial rheumatology assessment after referral was significantly longer for axSpA than RA patients. Assessment delays improved modestly over the period of observation; however, concerted effort will be required if the gap between RA and axSpA is to be narrowed further.

In RA, treatment delay by a matter of weeks is associated with erosive progression, reduced chance of remission and worse HAQ-DI trajectories [15]. There is a growing body of evidence that delayed axSpA diagnosis is associated with worse clinical, humanistic and economic outcomes [4], while treatment with TNF inhibitors has been shown to improve clinical outcomes and radiographic progression more effectively when commenced earlier in the disease process [5]. This has given rise to proposals for a ‘treat-to-target’ approach in axSpA, similar to that seen in RA and other chronic health conditions [16].

A major contributory factor to diagnostic delay in axSpA is poor recognition of key clinical features by healthcare professionals in primary and secondary care [17]. Multipartnership initiatives through the National Axial Spondyloarthritis Society (NASS) and British Society for Spondyloarthritis (BRITSpA), such as the Aspiring to Excellence programme [18], have made diagnostic delay a key learning outcome in their educational and service development activities. We note relatively few referrals from ophthalmology in the NEIAA dataset, mirroring the findings of previous axSpA surveys [19]. Several studies have identified a significant burden of undiagnosed axSpA in acute anterior uveitis [20, 21]; the one study estimated a minimum axSpA prevalence of 20.2% in a cohort of acute anterior uveitis patients, of whom one-quarter were previously undiagnosed [20]. Different screening tools have been proposed in this context: the Dublin Uveitis Evaluation Tool (DUET) recommends referral in patients who are HLA-B27 positive; SENTINEL goes one step further, recommending referral in patients <45 years of age with a history of chronic back pain, regardless of HLA-B27 status [21, 22].

Our finding that male gender associated with longer symptom durations than female gender in axSpA contrasts with what has been observed in other studies. Redeker *et al.* [2] demonstrated an association between female sex and longer diagnostic delay in axSpA, while a meta-analysis by Jovaní *et al.* [23] reported a mean diagnostic delay of 6.5 years for men and 8.8 years for women. It is possible that our finding is a chance observation or artefact due to selection effect. However, it may represent a true observation, arising from different clinical phenotypes between male and female axSpA patients in this cohort of newly referred patients. In contrast to many previous studies, the NEIAA does not restrict enrolment to radiographic or non-radiographic axSpA. Just under half of axSpA patients in the NEIAA had radiographic changes present, compared with 86% with MRI changes of sacroiliitis or SpA. A high proportion of non-radiographic axSpA, improved access to MRI and increasing awareness of axSpA as a condition of both women and men may have shifted the balance in gender-related diagnostic delay in this contemporaneous cohort of patients.

Of patients deemed eligible for follow-up in an EIA pathway, disease-specific education was provided to fewer axSpA than RA patients and RA patients reported a better understanding of their condition. This likely reflects a relative lack of specialist clinics for SpA patients. In a survey of 83 rheumatology departments by Derakhshan *et al.* [19], only 52% reported being able to offer additional dedicated patient education programmes for axSpA patients.

Interestingly, axSpA-EIA patients had better baseline HAQ-DI scores than RA patients but similar MSK-HQ scores. HAQ-DI is more upper limb–centric than MSK-HQ and, as such, may underrepresent disability and functional impairment in patients with predominantly axial symptoms. Our data on MSK-HQ scores show that the functional impact of axSpA is no less than for RA. The longer an individual is work impaired, the lower the likelihood that they will regain full work productivity, whereas prompt control of disease activity associates with large improvements in work productivity in early axSpA [24]. In our analyses, axSpA patients were more likely to be in paid work than RA patients. In addition to age and gender differences between the cohorts, other contributory factors to observed differences in work participation may include the typically more aggressive onset of symptoms in RA than axSpA, with higher TJCs and SJCs impacting on work involving manual dexterity. The male predominance and younger age of axSpA patients relative to RA is also likely to have contributed to differences in other characteristics, including higher smoking rates [25, 26].

Of note, the number of axSpA patients in the NEIAA was less than would be expected for disease prevalence in the UK population. A minimum prevalence of 0.3% (95% CI 0.13, 0.48) has been estimated using Assessment of SpondyloArthritis international Society classification criteria [27]. The NEIAA was designed to capture the referral of patients with any new inflammatory arthritis, including axSpA. While some centres interpreted this to mean patients with rheumatoid-pattern disease, other centres used a broader interpretation, reflected in the relative lack of objectively tender or swollen joints at baseline in axSpA-EIA patients in this cohort. An important limitation of this analysis is that we must acknowledge incomplete data capture. It is possible that centres with more robust pathways for inflammatory spinal disease may have been able to contribute more data to the audit, which, if true, could have led to an underestimation of treatment delay. In general, the underrepresentation of axSpA in the NEIAA reflects a trend towards poorer focus and resource allocation for axSpA compared with RA. In 2018 it was reported that 58% of NHS Trusts had a designated SpA service [19], whereas EIA clinics, which focus on RA and peripheral arthritis, are available in 77% of NHS Trusts [10].

Our study has a number of limitations. A broad definition was used by the NEIAA to define eligibility for EIA follow-up, interpretation of which will have differed between clinicians and centres; these selection effects must be considered when drawing inferences between EIA cohorts in these analyses. Several variables had missing data. Unlike for RA, conventional axSpA disease activity measures (BASDAI, ASDAS) were not available, as collection of these measures was not a core aim of the NEIAA. Data on NSAID or biologic DMARD use were also lacking. Future iterations of the NEIAA could be extended to include these important aspects. Fewer axSpA patients were HLA-B27 positive than might be expected; this might raise concerns about the validity of the diagnosis in some cases, although HLA-B27 positivity is not necessary for a diagnosis if other clinical criteria are met.

As a community, we have learned about the benefits of prompt diagnosis and treatment from across the spectrum of autoimmune diseases. Findings from the NEIAA highlight a subpopulation of patients with rheumatic disease on whom we need to focus more attention. We need to establish parity in relation to timely recognition, referral for assessment and patient education.

*Funding*: The National Early Inflammatory Arthritis Audit is commissioned by the Healthcare Quality Improvement Partnership, funded by NHS England and NHS Improvement and the Welsh government, and carried out by the British Society for Rheumatology, King’s College London and Net Solving. M.D.R. receives funding from the National Institute for Health Research as a Doctoral Fellow.

*Disclosure* *statement*: J.B.G. has received honoraria from AbbVie, Celgene, Chugai, Gilead, Janssen, Eli Lilly, Pfizer, Roche and UCB. M.Y. has received honoraria from UCB and AbbVie. M.D.R. has received honoraria and educational grants from Pfizer and UCB. K.G. has received consulting/speaker fees, research or institutional support and educational grants from AbbVie, Biogen, Celgene, Celltrion, Janssen, Eli Lilly, Novartis, Pfizer, Roche and UCB. R.S. has received consulting/speaker fees, research or institutional support and educational grants from AbbVie, Biogen, Celgene, Eli Lilly, MSD, Novartis, Pfizer, Roche and UCB. The other authors declare no conflicts of interest.

## Data availability statement

Data are available upon reasonable request by any qualified researchers who engage in rigorous, independent scientific research, and will be provided following review and approval of a research proposal and Statistical Analysis Plan (SAP) and execution of a Data Sharing Agreement (DSA). All data relevant to the study are included in the article.

## Supplementary data

Supplementary data are available at *Rheumatology* online.

## Supplementary Material

keab428_supplementary_data
