# Corrigendum to: Diagnostic delay is common for patients with axial spondyloarthritis: results from the National Early Inflammatory Arthritis Audit

**DOI:** 10.1093/rheumatology/keab665

**Published:** 2021-10-29

**Authors:** Mark D Russell, Fiona Coath, Mark Yates, Katie Bechman, Sam Norton, James B Galloway, Joanna Ledingham, Raj Sengupta, Karl Gaffney

**Affiliations:** Centre for Rheumatic Diseases, King’s College London, London; Rheumatology Department, Norfolk and Norwich University Hospital, Norwich; Centre for Rheumatic Diseases, King’s College London, London; Centre for Rheumatic Diseases, King’s College London, London; Centre for Rheumatic Diseases, King’s College London, London; Centre for Rheumatic Diseases, King’s College London, London; Rheumatology Department, Portsmouth Hospitals University NHS Trust, Portsmouth; Department of Rheumatology, Royal National Hospital for Rheumatic Diseases, Bath, UK; Rheumatology Department, Norfolk and Norwich University Hospital, Norwich

Rheumatology 2021;

doi:https://doi.org/10.1093/rheumatology/keab428

In the originally published version of this manuscript, there was an error in the *y*-axis of Figure 1B. It should read “Proportion of patients assessed within 3 weeks” instead of “Proportion of patients assessed within 3 months”. This error has been corrected online and in print.

