# Supplementary material for: Diagnostic delay is common for patients with axial spondyloarthritis: results from the National Early Inflammatory Arthritis Audit
Source: Rheumatology (Oxford). 2021 May 12;61(2):734–42. doi: 10.1093/rheumatology/keab428 (PMC8824413; doi:10.1093/rheumatology/keab428)
Supplement: keab428_supplementary_data [file keab428_supplementary_data.zip › rhe-21-0163-File004.docx]

**Supplementary Figure S1: Comparison of disease burden across individual domains of MSK-HQ in EIA-eligible patients with axSpA and RA**

Needing help

Pain/stiffness day

Confidence in managing symptoms

Understanding of your condition and treatment

Social activities and hobbies

Physical activity levels

axSpA

RA

Sleep

Fatigue/low energy

Emotional well-being

Overall impact

Pain/stiffness night

Walking

Washing/dressing

Work/daily routine

1

2

3

4

Radar plot of disease burden across the individual domains of the Musculoskeletal Health Questionnaire (MSK-HQ) in EIA-eligible patients with axSpA and RA. Each domain is scored by patients on a 0-4 scale, with higher scores representing lower impact on musculoskeletal health. For domains other than “Understanding of your condition and any current treatment” and “Confidence in being able to manage your symptoms”, a score of 0 represents severe impact of the symptoms on the domain, while a score of 4 represents no impact. For the domains “Understanding of your condition and any current treatment” and “Confidence in being able to manage your symptoms”, a score of 0 represents no understanding/confidence, while a score of 4 represents complete understanding/confidence. Further information on MSK-HQ is available in the glossary in Supplementary Material and from https://www.versusarthritis.org/media/7833/msk-hq-2018.pdf.

**Supplementary Table S1. Baseline characteristics of EIA-eligible vs. EIA-ineligible axSpA patients**.

|  | **Total** | **EIA-eligible axSpA** | **EIA-ineligible axSpA** |
| --- | --- | --- | --- |
|  | **N=762** | **N=222** | **N=540** |
| Age, years | 38 (30,49) | 37 (30,48) | 38 (30,49) |
| Gender |  |  |  |
| Female | 307 (40%) | 90 (40%) | 217 (40%) |
| Male | 455 (60%) | 132 (60%) | 323 (60%) |
| Ethnicity |  |  |  |
| White | 655 (86%) | 190 (86%) | 465 (86%) |
| Black British/African/Caribbean | 11 (1%) | 5 (2%) | 6 (1%) |
| Asian/Asian British | 54 (7%) | 16 (7%) | 38 (7%) |
| Mixed/Other Ethnic Groups | 39 (5%) | 10 (5%) | 29 (5%) |
| Not known | 3 | 1 | 2 |
| Smoking status |  |  |  |
| Current smoker | 178 (26%) | 50 (26%) | 128 (26%) |
| Ex-smoker | 149 (22%) | 43 (22%) | 106 (22%) |
| Never smoked | 363 (53%) | 103 (53%) | 260 (53%) |
| Not known | 72 | 26 | 46 |
| Within Most Deprived IMD Quintile |  |  |  |
| No | 555 (81%) | 175 (84%) | 380 (79%) |
| Yes | 132 (19%) | 34 (16%) | 98 (21%) |
| Not known | 75 | 13 | 62 |
| Duration of symptoms |  |  |  |
| <1 month | 15 (2%) | 6 (3%) | 9 (2%) |
| 1-3 months | 64 (8%) | 24 (11%) | 40 (7%) |
| 3-6 months | 74 (10%) | 23 (10%) | 51 (10%) |
| 6-12 months | 110 (15%) | 38 (17%) | 72 (13%) |
| 1-5 years | 248 (33%) | 69 (31%) | 179 (33%) |
| 5-10 years | 116 (15%) | 32 (15%) | 84 (16%) |
| >10 years | 131 (17%) | 29 (13%) | 102 (19%) |
| Not known | 4 | 1 | 3 |
| Time to initial assessment, days | 36 (20,64) | 33 (19,59) | 37 (20,67) |
| Assessment within 3 weeks of referral |  |  |  |
| No | 532 (70%) | 149 (67%) | 383 (71%) |
| Yes | 226 (30%) | 72 (33%) | 154 (29%) |
| Not known | 4 | 1 | 3 |
| HLA-B27 result |  |  |  |
| Positive | 256 (60%) | 60 (55%) | 196 (61%) |
| Negative | 174 (40%) | 49 (45%) | 125 (39%) |
| Not known | 332 | 113 | 219 |
| Radiographic changes |  |  |  |
| No | 218 (54%) | 47 (49%) | 171 (56%) |
| Yes | 185 (46%) | 48 (51%) | 137 (44%) |
| Not known | 359 | 127 | 232 |
| MRI changes |  |  |  |
| No | 55 (14%) | 11 (11%) | 44 (14%) |
| Yes | 352 (86%) | 88 (89%) | 264 (86%) |
| Not known | 355 | 123 | 232 |

Data are presented as median (IQR) for continuous measures and n (%) for categorical measures. Missing data is shown, but not included within the percentages for each category. AxSpA patients without data on eligibility for EIA (n=22) were not included. axSpA: axial spondyloarthritis; EIA: early inflammatory arthritis; IMD: Index of multiple deprivation (for further information on IMD, see glossary in Supplementary Material); Radiographic changes: changes suggestive of sacroiliitis or spondyloarthritis on plain radiographs; MRI changes: changes suggestive of sacroiliitis or spondyloarthritis on MRI.

**Glossary of terms**

NHS: National Health Service – the publicly-funded healthcare system of the United Kingdom.

IMD: Index of Multiple Deprivation – the official measure of relative deprivation in local areas across the United Kingdom. IMD combines information from seven domains (income, employment, education, skills and training, health and disability, crime, housing and services barriers, living environment) [11]. IMD can be ranked from most (1) to least deprived (5) quintiles.

HAQ-DI: Health Assessment Questionnaire Disability Index – a 10-item questionnaire developed to measure disability. Scores range from 0-3, with higher scores indicating worse functional status [10].

MSK-HQ: Musculoskeletal Health Questionnaire – a 15-item questionnaire evaluating symptom impact; validated for use across several musculoskeletal health conditions. A score is calculated from 14 domains and ranges from 0 to 56, with higher scores indicating better musculoskeletal health [10, 12].

WPAI: Work Productivity and Activity Index – a measure of work status and impact. WPAI overall impairment (expressed as a percentage, from low to high impact) incorporates both absenteeism (numbers of hours of work missed as a percentage of total hours worked) and presenteeism (degree to which patients’ health affects their productivity at work, as a percentage) [10, 13].
