# Supplementary material for: Diagnostic delay is common for patients with axial spondyloarthritis: results from the National Early Inflammatory Arthritis Audit
Source: Rheumatology (Oxford). 2021 May 12;61(2):734–42. doi: 10.1093/rheumatology/keab428 (PMC8824413; doi:10.1093/rheumatology/keab428)
Supplement: keab428_supplementary_data [file keab428_supplementary_data.zip › rhe-21-0163-File005.docx]

Being asked to define the cohort of the patients that were deemed eligible for further follow-up, authors, unfortunately, did not succeed; in fact, in the revised version of the manuscript, the definition of “EIA-eligible” axSpA patients has become even fuzzier. The authors claim that “a subset of patients recruited to NEIAA are deemed eligible for more frequent follow-up within an EIA pathway by treating rheumatologist; this is at the discretion of the clinician, on the basis of history, physical examination and laboratory testing“. With such an unprecise definition, a reader can not get an understanding of how the decision was made, based on which criteria the patients were selected for the further follow-up, and whether such a decision was made in the same way by all centres, i.e. the group of the patients is homogenous. What exactly in history, physical examination and laboratory testing have driven the decision of a treating rheumatologist to include the patient as eligible for further follow-up or not is not mentioned specifically; therefore, it is highly likely that no strict definitions existed (as authors fail to provide them). Moreover, the authors claim that “Baseline characteristics were similar between EIA-eligible and EIA-ineligible axSpA patients”. Section “Disease activity scores at baseline and DMARD use by 3 months in EIA-eligible axSpA and RA patients“ of the results shows that the “EIA-eligible” axSpA patients had medians of 0 tender joints, 0 swollen joints (so, at least half of the patients had no arthritis), median CRP of 5 mg/l (upper border of normal range) and ESR of 8 mm/h (normal), which raises questions on how such patients may be deemed “EIA-eligible” based (as authors state) on the “physical examination and laboratory testing”. Altogether, this makes the reasoning and the way of separating this subgroup unclear and therefore the analyses performed in this subgroup, in particular, comparisons between “EIA-eligible” axSpA patients and RA patients, can not be deemed meaningful. Either a feasible description of the group should be provided or all comparisons involving “EIA-eligible” axSpA patients and RA patients should be removed completely

- Thank you for these helpful comments. We entirely agree that there are selection effects that will vary by centre in relation to who is enrolled for EIA follow-up versus who is not. However, we do not feel that this detracts from the overall message of our report, and unfortunately, we cannot change the wording of the eligibility criteria included within this nationally-approved audit. We would highlight to the reviewer that we are in no way trying to draw inferential comparisons between these two groups. The reality is that our aim is to describe the state of play for axial spondyloarthritis care in the NHS across England and Wales, and highlight the areas where there are discrepancies in care quality. For example, there appears to be lower levels of educational provision for axSpA patients compared to RA. This is an observation we can only draw upon by inclusion of the EIA follow-up cohort. Therefore, despite the limitations of design, selection bias, confounding, misclassification etc., we still feel that there is an important message to share. We have further clarified our description of the EIA population (with wording drawn directly from the clinician instructions in NEIAA), and have articulated the limitations more clearly in the discussion.

Results, page 7, lines 22-26: “38/83 (45.8%) axSpA-EIA patients with data available commenced a DMARD by 3 months; 12 commenced methotrexate, 8 commenced sulfasalazine, 5 commenced hydroxychloroquine, and 14 commenced other unspecified DMARDs for which further details were unavailable.” – numbers are still wrong, 12+8+5+14≠38.

- The difference in numbers reflect that patients could be started on more than one DMARD: data were available for 38 patients, in whom 39 (in total) DMARDs were commenced. We have updated the methods text to make this clear.

Discussion, page 8, lines 6-7: “We note relatively few referrals from ophthalmology in the NEIAA dataset” – in the results, authors, unfortunately, do not provide how many patients were referred by different sources. It would be meaningful to provide such data first (and interesting to see indeed), otherwise, the further speculations are hard to follow in the discussion, as no numbers are given

- This information is provided in results (paragraph 3).

Discussion, page 8, lines 18-29 “Our finding that male gender associated with longer symptom durations than female gender in axSpA…” – a study by Redeker that authors already refer to (Reference 2) can be mentioned here as well.

- Thank you for pointing this out – we have updated the discussion text to include this reference.
